# Effects of Hemodiafiltration Versus Hemodialysis on Uremic Toxins, Inflammatory Markers, Anemia, and Nutritional Parameters: A Systematic Review and Meta-Analysis

**DOI:** 10.3390/toxins18020086

**Published:** 2026-02-06

**Authors:** Wannasit Wathanavasin, Solos Jaturapisanukul, Preeyaporn Janwetchasil, Charat Thongprayoon, Wisit Cheungpasitporn, Tibor Fülöp

**Affiliations:** 1 Nephrology Unit, Department of Medicine, Charoenkrung Pracharak Hospital, Bangkok Metropolitan Administration, Bangkok 10120, Thailand; aui.pree@gmail.com; 2 Nephrology and Renal Replacement Therapy Division, Department of Internal Medicine, Faculty of Medicine Vajira Hospital, Navamindradhiraj University, Bangkok 10300, Thailand; solos@nmu.ac.th; 3 Division of Nephrology and Hypertension, Department of Medicine, Mayo Clinic, Rochester, MN 55901, USA; thongprayoon.charat@mayo.edu (C.T.); cheungpasitporn.wisit@mayo.edu (W.C.); 4 Medicine Service, Ralph H. Johnson VA Medical Center, Charleston, SC 29401, USA; 5 Department of Medicine, Division of Nephrology, Medical University of South Carolina, Charleston, SC 29425, USA

**Keywords:** hemodiafiltration, hemodialysis, uremic toxins, inflammation, anemia, nutrition

## Abstract

Hemodiafiltration (HDF) is increasingly used because of its enhanced theoretical clearance of diverse uremic toxins, particularly middle molecules and inflammatory cytokines, relative to conventional hemodialysis (HD), yet evidence on its biochemical benefits remains conflicting. Therefore, this meta-analysis was performed to evaluate the effects of HDF versus HD on uremic toxins, inflammation, anemia, and nutritional parameters. A systematic literature search was conducted using PubMed, Scopus, and the Cochrane Central Register of Controlled Trials to identify relevant studies. Only randomized controlled trials (RCTs) were included. Random-effects meta-analyses were performed to evaluate changes in the prespecified outcomes. Twenty-four RCTs involving 6072 dialysis patients were included. Compared with conventional HD, HDF was associated with significant reductions in serum phosphorus (weighted mean difference [WMD] −0.28 mg/dL; 95% CI −0.44 to −0.12) and β2-microglobulin (WMD −4.84 mg/dL; 95% CI −6.13 to −3.54). HDF also significantly reduced serum urea and C-reactive protein (CRP) levels, along with weekly erythropoietin requirements. Serum albumin levels were slightly but significantly lower in the HDF group than in the conventional HD group (WMD –0.06 g/dL; 95% CI −0.10 to −0.01); however, the clinical significance of such a difference remains uncertain. Higher convective volumes were identified as a key determinant of greater reductions in β2-microglobulin and CRP. Compared with conventional HD, HDF demonstrated superior reductions in several surrogate endpoints, including serum phosphorus, urea, β2-microglobulin, CRP, and weekly erythropoietin requirements. Reduced need for phosphate binders and anemia management may lower treatment-related costs.

## 1. Introduction

End-stage kidney disease (ESKD) remains a major global health problem, associated with high cardiovascular morbidity and mortality despite advances in kidney replacement therapy [1,2,3]. Hemodialysis (HD) is the most widely used modality and achieves solute removal predominantly through diffusion [4]. While HD effectively clears small, water-soluble solutes such as urea and electrolytes, it has limited capacity to remove middle-molecular-weight toxins and protein-bound uremic solutes. Retention of these toxins, including β_2_-microglobulin and protein-bound compounds such as indoxyl sulfate (IS) and p-cresyl sulfate (PCS), has been implicated in chronic inflammation, vascular dysfunction, and adverse clinical outcomes [5,6].

Hemodiafiltration (HDF) was developed to address these limitations by combining diffusive and convective transport mechanisms [7]. By using high-flux membranes and achieving higher convective volumes, HDF is theoretically capable of enhancing the clearance of larger uremic toxins that are poorly removed by diffusion alone [7,8]. Interest in HDF has increased following large randomized trials, suggesting a survival benefit with high-volume HDF compared with conventional high-flux HD [9,10,11,12]. However, the mechanisms underlying this potential benefit remain incompletely understood [13,14]. 

Evidence regarding the superiority of HDF over conventional HD in removing specific classes of uremic toxins has been inconsistent. Individual studies have reported variable effects on the clearance of phosphate [11,15], middle molecules [10,16,17], and protein-bound uremic toxins (PBUTs) [18]. With respect to PBUTs, despite the theoretical benefits of convective therapy, current evidence for enhanced clearance with HDF remains inconclusive [19], largely due to the limited number of available studies and short follow-up durations. Moreover, differences in convective volume, dialyzer membrane type, and substitution fluid modality contribute substantially to heterogeneity in reported outcomes, making interpretation of the existing literature challenging. Beyond the potential enhanced removal of larger middle molecules, uremic toxins, the use of ultrapure dialysate in HDF may attenuate inflammatory burden and improve anemia-related outcomes, potentially translating into better appetite and nutritional status [20].

Therefore, we conducted a systematic review and meta-analysis to compare the efficacy of HDF and HD in the removal of uremic toxins across a broad range of molecular weights. By synthesizing data from randomized and crossover studies, this analysis aims to clarify whether HDF provides a meaningful advantage over HD in uremic toxin clearance, inflammatory markers, anemia-related outcomes, and nutritional parameters, thereby informing the optimization of dialysis strategies for patients with ESKD.

## 2. Results

### 2.1. Search Results

**Figure 1:**
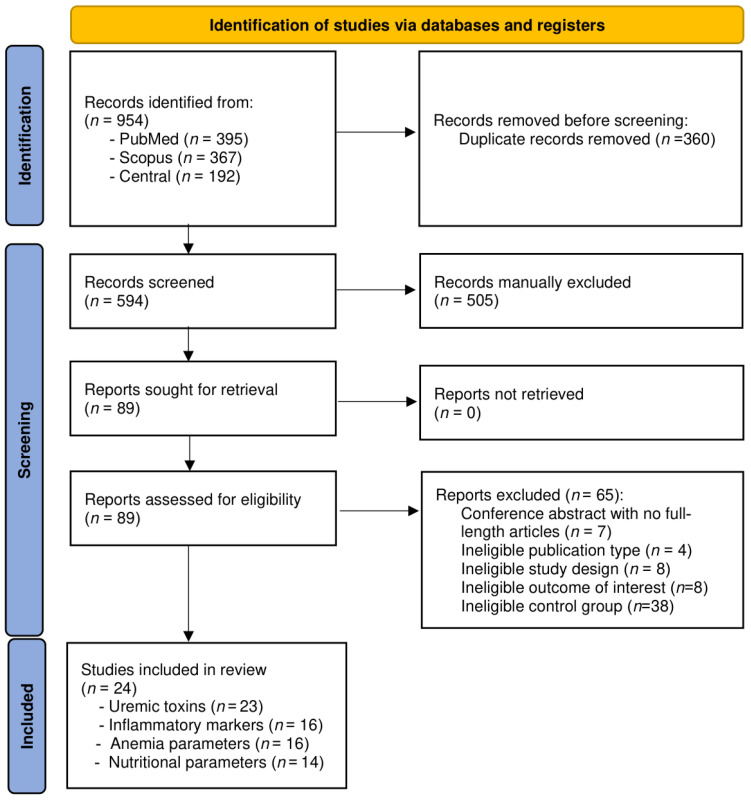
PRISMA 2020 flow diagram.

A total of 954 articles were identified and screened for relevance. Following the removal of 360 duplicate records, 594 titles and abstracts were assessed according to the predefined inclusion and exclusion criteria. Of these, 505 articles were excluded because they did not meet the inclusion criteria (e.g., non-randomized studies, reviews, conference abstracts, studies involving non-adult populations, or irrelevant outcomes). The remaining 89 articles were reviewed in full text, and 65 were excluded, with the reasons for exclusion outlined in Figure 1. Ultimately, 24 studies met the eligibility criteria (Figure 1), comprising 7 randomized crossover trials and 17 randomized parallel-arm controlled trials. Among these studies, 23 reported outcomes related to uremic toxins, 16 reported inflammatory and anemia-related markers, and 14 reported nutritional parameters. Among the crossover trials, six reported biochemical outcomes using combined data from both treatment periods, while one presented results separately for each study period.

### 2.2. Study Characteristics

The characteristics of the included studies are presented in Table 1. The studies covered a period from 2000 to 2023 and showed substantial variability in sample size, ranging from 15 to 1360 patients, with a total of 6072 ESKD patients included in this systematic review. Mean participant age ranged from 34.7 to 76.2 years, duration of dialysis from 10.7 to 83 months, and the prevalence of diabetes from 4.2% to 83%. The assessed outcomes, including uremic toxins and inflammatory, nutritional, and anemia-related parameters, as well as follow-up periods, are detailed in Table 1.

**Table 1:** Characteristics of the included studies.

No.	Author	Year	Country	Design	Sample Size	Age (Year)	Male (%)	DM (%)	Vintage (Month)	Outcomes of Interest				FU Time (Month)	Risk of Bias ^†^
										Uremic Toxins	Inflammatory Markers	Nutritional Markers	Anemia Parameters		
1	Wizemann V. [16]	2000	Germany	Parallel	44	60.5	56.8	18.2	NA	β2-MG	NA	Albumin	NA	24	High
2	Ward RA. [21]	2000	Germany	Parallel	45	56.8	64.4	NA	56.8	β2-MG, Pi, Urea	NA	NA	NA	12	High
3	Vaslaki LR. [22]	2005	Germany	Crossover	23	59	NA	NA	34	NA	IL-6, CRP	Albumin	NA	6	Some concerns
4	Schiffl H. [23]	2007	Germany	Crossover	76	62	55.3	19.7	45	β2-MG, Pi,	CRP	Albumin	Hb	24	Some concerns
5	Penne EL. [24]	2010	Netherlands, Canada, Norway	Parallel	493	66	62.1	23	26.5	Pi, PTH	NA	Albumin	NA	6	Low
6	Pedrini LA. [25]	2011	Italy	Crossover	69	59.6	69.6	NA	76	Urea, β2-MG, Pi, PTH	CRP	Albumin	Weekly ED	6	Low
7	Grooteman MP. [26]	2012	Netherlands, Canada, Norway	Parallel	714	64.1	62.3	23.8	34.8	β2-MG, Pi	CRP	Albumin	Hb	36	Low
8	Stefánsson BV. [27]	2012	Sweden	Crossover	20	60.6	70	35	NA	Urea, Pi, β2-MG	hs-CRP, IL-6	Albumin	Hb, ferritin	2	Low
9	Francisco RC. [28]	2012	Mexico	Parallel	24	34.7	33.3	NA	10.7	Pi	NA	Albumin	NA	3	Some concerns
10	Kantartzi K. [29]	2012	Greece	Crossover	24	62	79.2	4.2	31	Pi, PTH, β2-MG	CRP	Albumin	Hb, ferritin	6	Some concerns
11	Ok E. [11]	2013	Turkey	Parallel	782	56.5	58.9	34.7	57.9	Urea, Pi, PTH, β2-MG	hs-CRP	Albumin	Hb, ferritin, TSAT, weekly ED	24	Low
12	Maduell F. [10]	2013	Spain	Parallel	906	65.4	66.9	24.9	33	Pi, PTH, β2-MG	CRP	Albumin	Hb, ferritin, TSAT	36	Low
13	Bellien J. [30]	2014	France	Parallel	42	68.6	54.8	19	20.4	Pi, PTH, β2-MG	hs-CRP	Albumin	Hb	4	Low
14	den Hoedt CH. [31]	2014	Netherlands, Canada, Norway	Parallel	405	63.5	62	21	30.6	NA	hs-CRP, IL-6	Albumin	NA	36	Low
15	Karkar A. [32]	2015	Saudi Arabia	Parallel	72	54.6	42	33.8	51.5	Pi, PTH, β2-MG	NA	Albumin	Hb, ferritin, TSAT	24	High
16	Jiang X. [15]	2016	China	Parallel	48	56.8	58.3	NA	21.3	Urea, Pi, PTH, β2-MG	CRP, IL-6	NA	NA	3	High
17	Smith JR. [33]	2016	Scotland	Crossover	100	65	61	26	35	Pi, PTH	CRP	Albumin	Hb, ferritin	2	Low
18	Morena M. [34]	2017	France	Parallel	381	76.2	60.1	38.6	57.6	Pi, PTH, β2-MG	IL-6	NA	Hb, TSAT, ferritin	24	Some concerns
19	Cavallari C. [35]	2018	Italy	Parallel	30	64.6	72	28	83	Pi, PTH, β2-MG	CRP	NA	Hb, TSAT	9	Some concerns
20	Chu G. [36]	2019	Australia	Crossover	15	69.5	80	53.3	43.7	β2-MG	IL-6, hsCRP	NA	NA	2	Some concerns
21	van Gelder MK. [18]	2020	Netherlands	Parallel	80	62.9	56	21.3	22.7	PCS, IS	NA	NA	NA	6	Low
22	Pecoits-Filho R. [37]	2021	Brazil	Parallel	195	53	71.3	34.9	NA	Urea, Pi, PTH, β2-MG	NA	Albumin	Hb, TSAT, ferritin	6	Low
23	Kang A. [38]	2021	Australia	Parallel	124	65.5	55.6	35.5	41.2	β2-MG, Pi	NA	NA	Hb	48	Low
24	Blankestijn PJ. [12]	2023	Netherlands	Parallel	1360	62.4	62.9	35.4	40	Pi	CRP	NA	Hb	36	Low

Abbreviations: β2-MG, β2-microglobulin; CRP, C-reactive protein; DM, diabetes mellitus; ED, erythropoietin dose; FU, follow-up; Hb, hemoglobim; hs-CRP, high sensitivity C-reactive protein; IL-6, interleukin-6; IS, indoxyl sulfate; NA, not applicable; PCS, p-cresyl sulfate; Pi, phosphate; PTH, parathyroid hormone; and TSAT, transferrin saturation ^†^ Detailed risk-of-bias assessments are presented in the Methodological Quality section and in Figure 2A,B.

**Figure 2:**
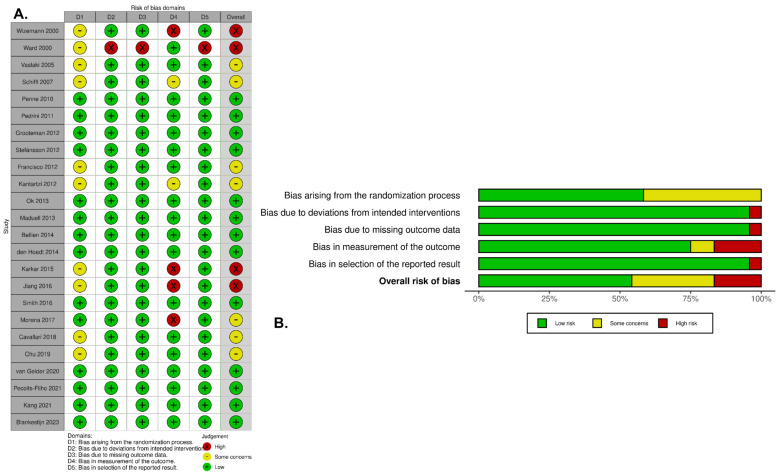
Risk-of-bias assessment of included RCTs: (**A**) traffic light plot and (**B**) weighted summary plot of the overall type of bias. The assessment tool evaluates domain-specific risk across five domains: D1, bias arising from the randomization process; D2, bias due to deviations from intended interventions; D3, bias due to missing outcome data; D4, bias in outcome measurement; and D5, bias in selection of the reported results [10,11,12,15,16,18,21,22,23,24,25,26,27,28,29,30,31,32,33,34,35,36,37,38].

### 2.3. Treatment Characteristics

Table 2 outlines the prescribed characteristics of HDF and HD treatments, including blood and dialysate flow rates, treatment frequency, dialyzer membrane, and session duration. For HDF, additional treatment-specific features, such as substitution mode and delivered/targeted convective volume, are also presented. In the HD group, high-flux dialyzers were used in 14 studies (58.3%), low-flux in 9 studies (37.5%), and mixed types in 1 study (4.2%). Within the HDF group, post-dilution mode was the most commonly used, as it was applied in 22 studies (91.7%).

**Table 2:** Overview of prescribed hemodiafiltration and hemodialysis treatment characteristics.

No.	Author	Hemodialfiltration Prescription						Hemodialysis Prescription				Duration (Hour/Session)
		Substitution Mode	BFR (mL/min)	DFR (mL/min)	Frequency	Dialyzer Membrane	Delivered Convective Volume (L/Session)	BFR (mL/min)	DFR (mL/min)	Frequency	Dialyzer Membrane	
1	Wizemann V. [16]	NA	400–500	100–200	NA	High flux	60 *	400–500	500	NA	Low flux	4.5
2	Ward RA. [21]	Post-dilution	281 ± 4	500	3/wk	High flux	21 ± 1	274 ± 4	500	3/wk	High flux	4.1–4.18
3	Vaslaki LR. [22]	Post-dilution	296 ± 5	500	3/wk	High flux	23.7	296 ± 5	500	3/wk	Low flux	4.2
4	Schiffl H. [23]	Post-dilution	250–350	500	3/wk	High flux	19.1	250–350	500	3/wk	High flux	4.23
5	Penne EL. [24]	Post-dilution	302–330	NA	3/wk	High flux	19.5 ± 4.3	299–309	NA	3/wk	Low flux	3.75–3.78
6	Pedrini LA. [25]	Mixed (Pre-, Post-, Mixed-dilution)	346 ± 35	616 ± 87	3/wk	High flux	Post-dilution 19.7 ± 4.2, Mixed-dilution 37.7 ± 4.8, Pre-dilution 46.3 ± 7.6	348 ± 37	507 ± 45	3/wk	Low flux	3.77
7	Grooteman MP. [26]	Post-dilution	300–400	NA	2–3/wk	High flux	20.7 ± 6	300–400	NA	2–3/wk	Low flux	3.77–3.81
8	Stefánsson BV. [27]	Post-dilution	310 ± 34	500	NA	High flux	24.4	312 ± 32	500	NA	Low flux	4.42–4.45
9	Francisco RC. [28]	Post-dilution	430	500	3/wk	High flux	18	400	500	3/wk	High flux	4
10	Kantartzi K. [29]	Post-dilution and Prepared bag	250–350	500–700	3/wk	High flux	Online-HDF 15–20 L/session, HDF 10 L/session	250–350	500–700	3/wk	Low flux	4
11	Ok E. [11]	Post-dilution	250–400	500	3/wk	High flux	17.2 ± 1.3	250–400	500	3/wk	High flux	4
12	Maduell F. [10]	Post-dilution	384–392	553–580	3/wk	High flux	23.7	367–380	531–560	3/wk	High flux 91.9%, Low flux 8.1%	3.93
13	Bellien J. [30]	Post-dilution	400	800	3/wk	High flux	26.6 ± 2.9	400	800	3/wk	High flux	4
14	den Hoedt CH. [31]	Post-dilution	305 ± 37	NA	2–3/wk	High flux	18.7	308 ± 35	NA	2–3/wk	Low flux	3.79
15	Karkar A. [32]	Post-dilution	331 ± 27	NA	3/wk	High flux	19.3 ± 2.1	328 ± 31	NA	3/wk	High flux	4
16	Jiang X. [15]	NA	250–300	500	3/wk	High flux	NA	250–300	500	3/wk	High flux	4–4.5
17	Smith JR. [33]	Post-dilution	313 ± 28	NA	3/wk	High flux	20.6 ± 4.6	315 ± 27	NA	3/wk	High flux	4.17
18	Morena M. [34]	Mixed (Post- (mainly), Pre-dilution)	350–400	500–600	3/wk	High flux	Post-dilution 22.48 ± 6.26, Pre-dilution 42.59 ± 16.38	350–400	500–600	3/wk	High flux	3.91–3.98
19	Cavallari C. [35]	Post-dilution	>250	500	3/wk	High flux	34.5 ± 4.2	>250	500	3/wk	High flux	4
20	Chu G. [36]	Post-dilution	NA	NA	3/wk	High flux	26.2 ± 3.8	NA	NA	3/wk	High flux	NA
21	van Gelder MK. [18]	Post-dilution	NA	NA	2–3/wk	High flux	17.3 ± 4.3	NA	NA	2–3/wk	Low flux	NA
22	Pecoits-Filho R. [37]	Post-dilution	NA	NA	NA	High flux	22 *	NA	NA	NA	High flux	4
23	Kang A. [38]	Post-dilution	304 ± 19	500	NA	High flux	24.7	300 ± 18	500	NA	High flux	14.8 hr/wk
24	Blankestijn PJ. [12]	Post-dilution	369 ± 54	NA	3/wk	High flux	25.3	367 ± 56	NA	3/wk	High flux	4

Abbreviations: BFR, blood flow rate; DFR dialysate flow rate; HDF, hemodiafiltration; and NA, not applicable. * Targeted convective volume.

### 2.4. Methodological Quality

Assessment using the Cochrane Risk of Bias 2 (RoB 2) tool classified thirteen studies as having a low risk of bias, seven studies as having some concerns, and four studies as presenting a high risk of bias. Figure 2 provides an overview of the judgments across individual RoB 2 domains for each included study. Overall, the included trials demonstrated low-to-moderate risk of bias (Figure 2A,B).

### 2.5. Effect of HDF Versus HD on Uremic Toxin Reduction (Table 3)

#### 2.5.1. Serum Urea

The mean baseline pre-dialysis serum urea concentration was 119.82 ± 43.54 mg/dL. A meta-analysis of six studies, including 1234 patients with ESKD, showed that HDF significantly reduced pre-dialysis serum urea compared with conventional HD (WMD −10.73 mg/dL; 95% CI −16.90 to −4.56; *p* < 0.01; I^2^ = 73.6%).

**Table 3:** Summary effects of hemodiafiltration versus conventional hemodialysis on biochemical parameters.

Outcomes	No. of Studies	No. of Patients			Baseline Mean Value (±SD)	Weighted Mean Difference (95% CI)	*p*-Values	I^2^
		Total	HDF	HD				
**Uremic toxins**								
Urea (mg/dL)	6	1234	620	614	119.82 (43.54)	−10.73 (−16.90 to −4.56)	<0.01	73.6
Phosphate (mg/dL)	18	4804	2423	2381	4.52 (1.98)	−0.28 (−0.44 to −0.12)	<0.01	92
Parathyroid hor-mone (pg/mL)	12	2914	1474	1440	273.67 (287.8)	+1.00 (−9.38 to 11.37)	0.85	5.4
β2-microglobulin (mg/dL)	16	3198	1616	1582	23.42 (14.8)	−4.84 (−6.13 to −3.54)	<0.01	95.5
P-cresyl sulfate (µmol/L)	1	80	39	41	NA	−3.9 (−12.53 to 4.73) *	0.85	NA
Indoxyl sulfate (µmol/L)	1	80	39	41	NA	−18.57 (−26.77 to −10.38) *	0.045	NA
**Inflammatory markers**								
C-reactive protein (mg/L)	12	3508	1802	1776	10.95 (19.36)	−0.94 (−1.53 to −0.35)	<0.01	79.8
Hs-CRP (mg/L)	3	864	433	431	16.91 (23.29)	−0.77 (−2.96 to 1.42)	0.49	0
IL-6 (pg/L)	7	978	487	491	26.48 (63.75)	−1.89 (−6.35 to 2.56)	0.40	87.9
**Anemia parameters**								
Hemoglobin (g/dL)	14	4563	2297	2266	11.61 (1.36)	0.06 (−0.06 to 0.18)	0.34	82.8
TSAT (%)	5	1229	624	605	28.48 (12.54)	−2.40 (−7.77 to 1.40)	0.38	95.3
Ferritin (µg/L)	9	2247	1133	1114	576.41 (545.31)	−41.80 (−134.77 to 51.17)	0.38	91.1
Weekly erythro-poietin dosage (units)	2	906	453	453	NA	−587.8 (−917.1 to −258.5)	<0.01	0
**Nutritional indicators**								
Albumin (g/dL)	15	3310	1667	1643	3.62 (2.17)	−0.06 (−0.10 to −0.01)	0.02	74.3

* Values are presented as mean change (95% CI). Abbreviation: NA, not applicable.

#### 2.5.2. Serum Phosphorus

**Figure 3:**
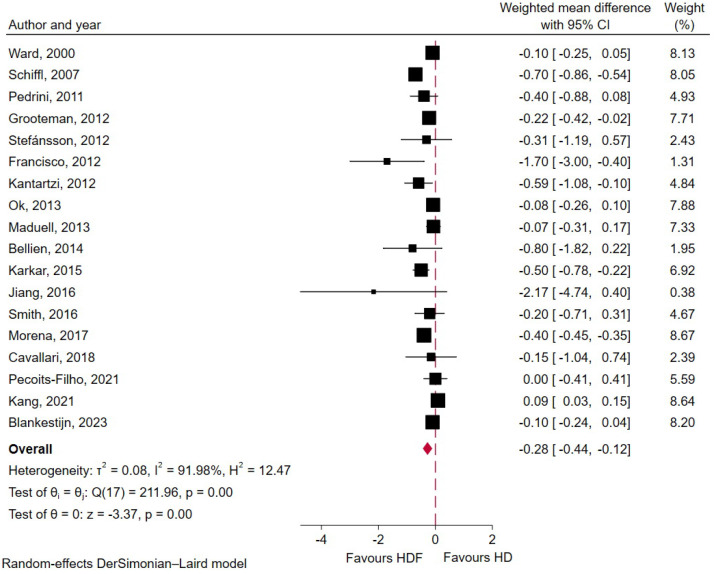
Forest plot displaying weighted mean difference in pre-dialysis serum phosphorous between HDF and conventional HD groups [10,11,12,15,21,23,25,26,27,28,29,30,32,33,34,35,37,38].

At baseline, pre-dialysis serum urea levels were 4.52 ± 1.98 mg/dL. Across 18 studies comprising 4804 patients with ESKD, HDF was associated with a statistically significant decrease in pre-dialysis serum phosphorus compared with conventional HD (WMD −0.28 mg/dL; 95% CI −0.44 to −0.12; *p* < 0.01; I^2^ = 92%, Figure 3). Subgroup analyses (Table S1) demonstrated a consistent effect across studies regardless of follow-up duration, dialyzer membrane type, substitution mode, or patient race (all *p* for interaction >0.05). However, crossover studies demonstrated a more pronounced reduction in serum phosphorous levels with HDF than parallel-design.

#### 2.5.3. Serum Parathyroid Hormone (PTH)

The mean baseline pre-dialysis serum PTH was 273.67 ± 287.8 pg/mL. A meta-analysis of 12 studies involving 2914 ESKD patients found that HDF did not significantly reduce pre-dialysis serum PTH compared with conventional HD (WMD +1.00 pg/mL; 95% CI −9.38 to 11.37; *p* = 0.85; I^2^ = 5.4%). Subgroup analyses (Table S2) demonstrated a consistent effect across studies regardless of study design, follow-up duration, dialyzer membrane type, substitution mode, or patient race (all *p* for interaction >0.05).

#### 2.5.4. Serum β2-Microglobulin

**Figure 4:**
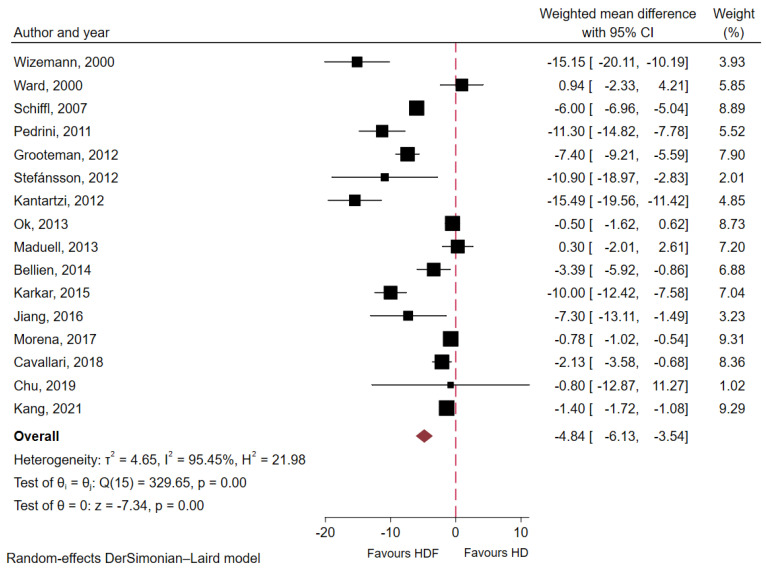
Forest plot displaying weighted mean difference in pre-dialysis serum β2-microglobulin between HDF and conventional HD groups [10,11,15,16,21,23,25,26,27,29,30,32,34,35,36,38].

At baseline, pre-dialysis serum β2-microglobulin level was 23.42 ± 14.8 mg/dL. Meta-analytic results from 16 studies including 3198 patients indicated that HDF significantly lowered pre-dialysis serum β2-microglobulin levels relative to conventional HD (WMD −4.84 mg/dL; 95% CI −6.13 to −3.54; *p* < 0.01; I^2^ = 95.5%, Figure 4). Subgroup analyses (Table S3) showed a consistent effect across studies regardless of substitution mode (*p* for interaction = 0.29). However, studies using a low-flux dialyzer membrane as the comparator demonstrated a significantly greater reduction in serum β2-microglobulin levels with HDF than those using a high-flux dialyzer (WMD −11.84 mg/dL; 95% CI −15.65 to −8.03 vs. −2.65 mg/dL; 95% CI −3.80 to −1.49; *p* for interaction <0.01).

#### 2.5.5. Serum p-Cresyl Sulfate (PCS)

In one study including 80 patients with ESKD, HDF did not significantly reduce pre-dialysis serum PCS compared with conventional HD, with a mean change of −3.9 µmol/L (95% CI −12.53 to 4.73) at six months of follow-up (*p* = 0.854).

#### 2.5.6. Serum Indoxyl Sulfate (IS)

In one study including 80 patients with ESKD, HDF significantly decreased pre-dialysis serum IS compared with conventional HD, with a mean change of −18.57 µmol/L (95% CI −26.77 to −10.83) after six months of follow-up (*p* = 0.045).

### 2.6. Effect of HDF Versus HD on Inflammatory Markers (Table 3)

#### 2.6.1. Serum C-Reactive Protein (CRP)

**Figure 5:**
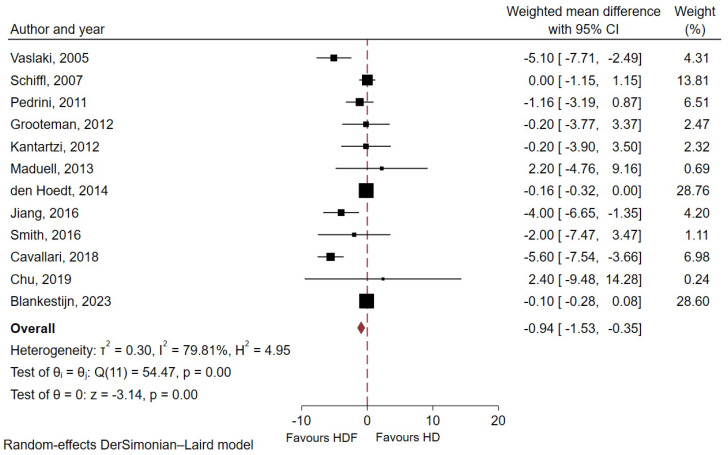
Forest plot displaying weighted mean difference in pre-dialysis serum C-reactive protein between HDF and conventional HD groups [10,12,15,22,23,25,26,29,31,33,35,36].

The mean baseline pre-dialysis serum CRP level was 10.95 ± 19.36 mg/L. Meta-analytic results from 12 studies including 3508 patients with ESKD showed that HDF significantly reduced pre-dialysis serum CRP compared with conventional HD (WMD −0.94 mg/L; 95% CI −1.53 to −0.35; *p* < 0.01; I^2^ = 79.8%, Figure 5). Subgroup analyses (Table S4) showed a consistent effect across study design, dialyzer membrane type, or substitution mode (all *p* for interaction >0.05). Notably, studies with shorter follow-up periods (≤6 months) showed a significantly greater reduction in serum CRP levels with HDF compared to studies with longer follow-up (>6 months) (WMD −2.54 mg/dL; 95% CI −4.39 to −0.70 vs. −0.45 mg/dL; 95% CI −0.99 to 0.09; *p* for interaction = 0.03).

#### 2.6.2. Serum High Sensitivity C-Reactive Protein (hs-CRP)

At baseline, pre-dialysis serum hs-CRP level was 16.91 ± 23.29 mg/L. A meta-analysis of three studies including 864 patients with ESKD demonstrated that HDF did not significantly reduce pre-dialysis serum hs-CRP compared with conventional HD (WMD −0.77 mg/L; 95% CI −2.96 to 1.42; *p* = 0.49; I^2^ = 0%).

#### 2.6.3. Serum Interleukin-6 (IL-6)

The mean baseline pre-dialysis serum IL-6 level was 26.48 ± 63.75 pg/L. Meta-analytic results from seven studies including 978 patients with ESKD demonstrated that HDF did not significantly reduce pre-dialysis serum IL-6 compared with conventional HD (WMD −1.89 pg/L; 95% CI −6.35 to 2.56; *p* = 0.40; I^2^ = 87.9%).

### 2.7. Effect of HDF Versus HD on Anemia Parameters (Table 3)

#### 2.7.1. Serum Hemoglobin

At baseline, pre-dialysis serum hemoglobin level was 11.61 ± 1.36 g/dL. In a meta-analysis of 14 studies comprising 4463 ESKD patients, pre-dialysis serum hemoglobin levels were not significantly increased by HDF compared with conventional HD (WMD +0.06 g/dL; 95% CI −0.06 to 0.18; *p* = 0.34; I^2^ = 82.8%). Subgroup analyses (Table S5) demonstrated a consistent effect across studies regardless of study design, follow-up duration, dialyzer membrane type, or substitution mode (all *p* for interaction >0.05).

#### 2.7.2. Serum Transferrin Saturation (TSAT)

The mean baseline pre-dialysis serum TSAT was 28.48 ± 12.54%. Meta-analytic results from five studies involving 1229 patients with ESKD showed no significant difference in pre-dialysis serum transferrin saturation between HDF and conventional HD (WMD −2.40%; 95% CI −7.77 to 1.40; *p* = 0.38; I^2^ = 95.3%).

#### 2.7.3. Serum Ferritin

At baseline, pre-dialysis serum ferritin level was 576.41 ± 545.31 µg/L. A meta-analysis of nine studies involving 2247 ESKD patients showed that HDF did not significantly affect pre-dialysis serum transferrin saturation compared with conventional HD (WMD −41.80 µg/L; 95% CI −134.77 to 51.17; *p* = 0.38; I^2^ = 91.1%).

#### 2.7.4. Weekly Erythropoietin Dosage

Across two studies including 906 ESKD patients, HDF was associated with a statistically significant reduction in weekly erythropoietin dosage compared with conventional HD (WMD −587.8 units; 95% CI −917.1 to −258.5; *p* < 0.01; I^2^ = 0%).

### 2.8. Effect of HDF Versus HD on Nutritional Marker

#### Serum Albumin

**Figure 6:**
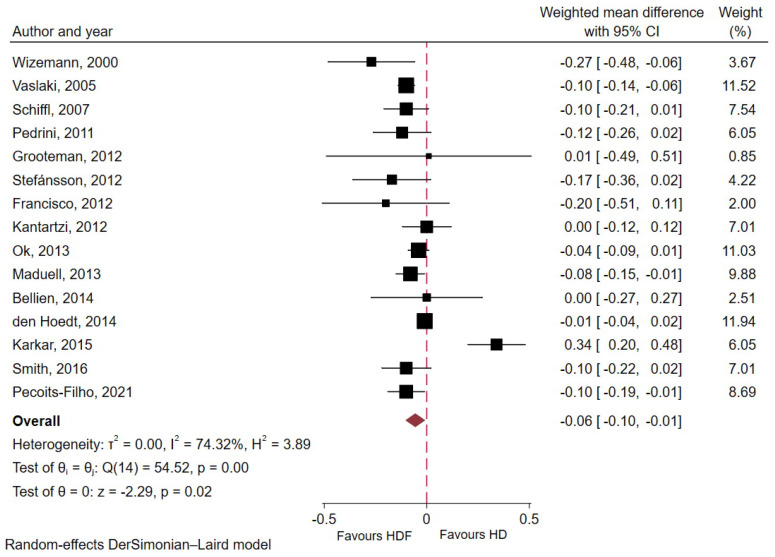
Forest plot displaying weighted mean difference in pre-dialysis serum albumin between HDF and conventional HD groups [10,11,16,22,23,25,26,27,28,29,30,31,32,33,37].

The mean baseline pre-dialysis serum albumin was 3.62 ± 2.17 g/dL. In a meta-analysis of 15 studies comprising 3310 ESKD patients, pre-dialysis serum albumin levels were significantly reduced in the HDF group compared with the conventional HD group (WMD −0.06 g/dL; 95% CI −0.10 to −0.01; *p* = 0.02; I^2^ = 74.3%, Figure 6). Subgroup analyses (Table S6) demonstrated a consistent effect across studies regardless of study design, follow-up duration, dialyzer membrane type, or substitution mode (all p for interaction >0.05).

### 2.9. Meta-Regression

**Figure 7:**
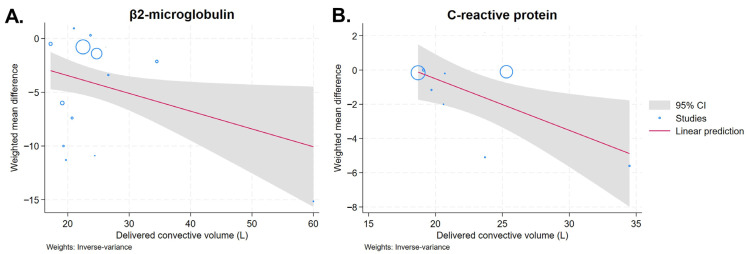
Meta-regression examining the association between convective volume and the reduction effect of HDF versus HD on serum β2-microglobulin (**A**) and C-reactive protein (**B**).

Meta-regression analyses were conducted for outcomes reported in more than 10 studies, including serum β2-microglobulin, phosphorus, parathyroid hormone, CRP, and serum albumin. These analyses showed no significant associations between treatment effects and participant age, sample size, convective volume, dialysis vintage, or publication year for serum parathyroid hormone and albumin levels (Table S7). However, greater reductions in serum β2-microglobulin (β = −017; *p* = 0.033; R^2^ = 0%) and CRP (β = −0.30; *p* = 0.017; R^2^ = 0%) were significantly associated with higher convective volumes prescribed during HDF (Figure 7A,B). Additionally, higher reductions in serum CRP (β = −0.045; *p* = 0.033; R^2^ = 0%) were significantly associated with patients with longer dialysis vintage (Table S7).

### 2.10. Assessment of Publication Bias

Publication bias was evaluated using visual inspection of funnel plots in combination with Egger’s regression test. Funnel plots for the effects of HDF on serum albumin, ferritin, hemo-globin, and IL-6 appeared symmetrical (Figures S1–S4), suggesting no evidence of publication bias; this was further supported by non-significant Egger’s test results (*p* > 0.05). In contrast, funnel plots for serum β2-microglobulin, CRP, and phosphorus showed asymmetry (Figures S5–S7), with Egger’s test indicating significant publication bias (*p* < 0.05).

## 3. Discussion

Our systematic review and meta-analysis evaluated the effects of HDF versus HD on uremic toxins and key biochemical parameters across 24 RCTs involving 6072 ESKD patients. Overall, HDF was associated with greater improvements in several biochemical parameters, including serum phosphorus, urea, β2-microglobulin, and CRP, along with significantly lower weekly erythropoietin requirements. These findings support the role of convective transport in enhancing the removal of middle-molecular-weight solutes beyond diffusion alone [4,6,7,8], although the extent of benefit varied according to toxin type, markers of inflammation, and anemia parameters. However, serum albumin levels were significantly lower in the HDF group than in the conventional HD group. Higher convective volumes were identified as a key determinant of greater reductions in β2-microglobulin and CRP. It should be noted that our analysis utilized pre-dialysis plasma levels as indicators of steady-state toxin exposure, which are more closely associated with long-term outcomes than intradialytic pre–post reductions that are influenced by rebound, redistribution, and sampling variability.

One of the most robust findings was the superior reduction in serum β2-microglobulin with HDF. Across 16 RCTs including more than 3000 ESKD patients, HDF was consistently associated with lower β2-microglobulin levels, despite substantial heterogeneity (Table 3). Larger effects were observed in crossover studies, shorter follow-up periods, and when low-flux HD was used as the comparator, underscoring the influence of study design and baseline membrane performance. Although the magnitude of benefit was reduced when HDF was compared with high-flux HD, the advantage remained, indicating that convective transport provides additional clearance beyond membrane permeability alone. Meta-regression showed that a higher effluent dose was independently associated with greater reductions in β2-microglobulin, supporting a dose-dependent effect (Figure 7A). β2-microglobulin is a well-established middle-molecular-weight marker, and its enhanced removal reflects a defining feature of HDF, which is characterized by the ability to achieve a β2-microglobulin sieving coefficient greater than 0.6 [39]. Evidence from several large randomized trials, including ESHOL [10], indicates that high-volume online HDF is associated with reduced β2-microglobulin accumulation and improved survival, supporting the clinical relevance of enhanced β2-microglobulin removal as an indicator of treatment benefit. In contrast, no consistent effect was observed for PTH, suggesting that endocrine regulation and bone turnover play a greater role than extracorporeal clearance in determining circulating hormone levels [40,41].

For small solutes, HDF was associated with modest but statistically significant reductions in serum urea and phosphate compared with HD. Given that small solutes are efficiently cleared by diffusion [6], these differences likely reflect greater overall solute flux with HDF rather than a fundamental difference in clearance mechanism. Increased effective membrane surface area, higher blood and dialysate flow rates, and the contribution of convective transport may enhance mass transfer [42], particularly in high-volume HDF. However, with modern HD already achieving high diffusive efficiency, further reductions in small-solute concentrations are subject to diminishing returns and are unlikely to be clinically meaningful. Notably, the modest 0.28 mg/dL phosphate reduction observed with HDF from a baseline of 4.52 mg/dL (Table 3) may have limited clinical relevance. Our analyses identified study design as a contributor to the high heterogeneity in serum phosphorus outcomes (Table S1). Crossover studies reported a larger reduction in serum phosphorus with HDF compared with parallel-group trials. This likely reflects the crossover design, in which participants act as their own controls, reducing inter-individual variability and increasing statistical efficiency. As a result, treatment effects—particularly for outcomes with substantial biological variability, such as serum phosphorus—may appear more pronounced. In addition to dialytic clearance, serum phosphate levels are influenced by multiple factors, including dietary intake, residual kidney function, and concomitant therapies, including phosphate binders, vitamin D analogs, and calcimimetics [43]. Importantly, serum phosphorus functions as a “dual marker,” reflecting both nutritional status and phosphate burden. In treatment modalities that improve appetite, reductions in serum phosphorus alone may not adequately capture broader clinical benefits, including effects on survival. Accordingly, the variability in these factors at baseline and during the study period across different studies may have contributed to the high heterogeneity observed.

Evidence regarding protein-bound uremic toxins remains limited. Only one RCT [18] assessed indoxyl sulfate and p-cresyl sulfate and found a non-significant reduction in PCS and a borderline significant reduction in IS, limiting definitive interpretation. These findings contrast with those of Duval-Sabatier et al. [44], who reported a significant reduction in PCS levels using both pre- and post-dilution HDF compared with conventional HD, although no significant difference in IS reduction was observed. Similarly, Tiranathanagul et al. [45] demonstrated that the use of super high-flux HD versus super high-flux HDF resulted in no significant difference in IS reduction. While HDF may theoretically enhance the removal of protein-bound uremic toxins through convective transport, both diffusion and convection appear insufficient to achieve significant clearance. This limitation is primarily due to the high binding affinity of these toxins to proteins, forming large molecular complexes that exceed the pore size of standard dialysis membranes. Given the small sample size of the available studies, the efficacy of HDF in reducing protein-bound uremic toxins remains inconclusive and warrants further investigation.

Given their link to cardiovascular disease [5,6], the limited data on protein-bound uremic toxins remain a key gap in the literature.

HDF was associated with lower serum CRP levels, suggesting a potential reduction in systemic inflammation. Greater effects in shorter-duration studies may reflect early improvements related to membrane biocompatibility [46] or reduced inflammatory exposure, possibly associated with the use of ultrapure dialysate [47]. However, no consistent differences were observed for IL-6 or hs-CRP, and heterogeneity remained substantial, indicating that chronic inflammation in dialysis patients is influenced by factors beyond solute removal, such as vascular access type [48], dietary quality, and individual nutritional status [49,50,51]. Moreover, the small sample sizes in IL-6 and hs-CRP analyses may have limited the statistical power to detect significant differences. Although anemia-related parameters, including hemoglobin, ferritin, and transferrin saturation, were similar between the two modalities, our study found that HDF was associated with significantly lower weekly erythropoietin requirements, approximately 600 units/week, compared with conventional HD (Table 3). This effect may be attributed to HDF’s potential to reduce inflammation and enhance the clearance of middle-molecule erythropoietic inhibitors, thereby improving iron utilization and enhancing the response to erythropoietin therapy.

Although serum albumin levels were slightly lower with HDF (WMD −0.06 g/dL; 95% CI −0.10 to −0.01; Figure 6) compared to a mean baseline pre-dialysis serum albumin level of 3.62 g/dL, the absolute difference was small and unlikely to be clinically relevant. Moreover, this borderline significant effect was mainly observed in crossover studies and short-term follow-up (Table S6), suggesting transient changes rather than true or sustained decline in nutritional status. Interestingly, studies by Tashiro [52] and Okada et al. [53] consistently reported that higher estimated albumin leakage (EAL) in patients undergoing online HDF was associated with improved survival. These findings suggest that incidental removal of protein-bound uremic toxins with albumin loss may underlie the observed survival benefit. Furthermore, high EAL accompanied by mild-to-moderate hypoalbuminemia did not appear to adversely affect survival outcomes in this population, particularly when albumin production is preserved. Convective albumin loss may therefore serve as a net clearance mechanism for protein-bound uremic toxins, especially in anuric patients. Variability across studies likely reflects differences in dialyzer characteristics and treatment parameters, including membrane permeability, ultrafiltration coefficient, and convective volume. In addition, short-term serum albumin measurements may not reliably reflect dialytic albumin loss, as intravascular redistribution and fluid removal during dialysis can influence measured levels. Enhanced clearance of inflammatory mediators with HDF may also reduce inflammation-related albumin catabolism [54]. Consistent with this interpretation, meta-regression did not identify effluent dose as a determinant of albumin reduction (Table S7). Okada et al. [55] confirmed that ESKD patients with normal to mildly reduced albumin levels and high albumin loss during HDF experienced improved survival, regardless of protein-energy wasting (PEW) or inflammation status. Overall, albumin leakage or serum albumin levels appeared to have minimal impact on clinical outcomes, particularly mortality, in patients without PEW or significant inflammation, supporting the safety of appropriately prescribed HDF.

The strengths of our meta-analysis include its large sample size and the demonstration of HDF’s beneficial effects on several surrogate markers, including uremic toxins, inflammatory markers, and the potential to reduce erythropoietin requirements in anemia management. To ensure the accuracy of our results, we included only RCTs and used exclusively valid, non-imputed data. In addition, our analysis addressed gaps from previous meta-analyses [56,57] by providing key surrogate outcomes that may help explain the mechanisms underlying HDF’s clinical benefits. We also explored potential sources of heterogeneity, including study design, substitution mode, convective volume, intervention duration, and dialyzer type in the comparator group, offering a comprehensive assessment of factors influencing outcomes.

Several limitations should also be acknowledged. Substantial heterogeneity observed in most surrogate outcomes may have been influenced by several factors discussed previously, potentially affecting the accuracy of pooled estimates. Although we performed subgroup and meta-regression analyses, several important factors, such as concomitant medications, dietary intake, and nutritional status, could not be evaluated due to the limited availability of trials addressing specific subgroups. Furthermore, definitions and reporting of convective dose and effective convective dose varied considerably among studies; some trials reported prescribed convective volumes, whereas others reported delivered or total substitution volumes, and few provided standardized measures of effective convection. This inconsistency likely limited our ability to directly attribute observed biochemical effects to the convective component of HDF and may have contributed to residual heterogeneity. Additionally, many of the included studies were small, of short duration, and varied in methodological quality. Data on certain uremic toxins, particularly protein-bound toxins, were limited, restricting conclusions in this important area. Moreover, most biochemical outcome data from crossover studies were analyzed as parallel-group data without accounting for intervention period effects, which may have biased the pooled effect estimates. Finally, publication bias was observed for β2-microglobulin, CRP, and phosphorus, as indicated by funnel plot asymmetry and significant Egger’s tests. This suggests that small studies with non-significant results may be underrepresented, potentially leading to an overestimation of effect sizes.

From a clinical perspective, our results suggest that HDF offers a meaningful advantage over HD, primarily in the removal of middle-molecular-weight uremic toxins (i.e., β2-microglobulin), potentially alleviating inflammation, particularly when high convective volumes are achieved. This supports the preferential use of high-volume HDF in patients at risk for complications related to middle-molecule retention, such as dialysis-related amyloidosis [6,58,59] or chronic inflammation [6,59]. Nonetheless, expectations for improvements in protein-bound toxin clearance and CKD-MBD markers other than phosphorus should be tempered. Further rigorous studies are required to assess whether enhanced removal of solutes, particularly protein-bound uremic toxins, translates into meaningful long-term outcomes. In addition, the increasing use of newly introduced medium cut-off (MCO) membranes, developed to enhance middle-molecule clearance during conventional HD [60], may attenuate differences in outcomes between HD and HDF. Nevertheless, robust clinical trial data directly comparing HD using MCO membranes with HDF remain limited, and further adequately powered studies are required to determine whether HD with MCO membranes can achieve clinical benefits comparable to those of HDF.

## 4. Conclusions

In summary, HDF provides superior reductions in several surrogate endpoints, including serum phosphorus, urea, β2-microglobulin, CRP, and weekly erythropoietin requirements compared with conventional HD. However, substantial heterogeneity—particularly for phosphorus, β2-microglobulin, and CRP—warrants cautious interpretation of these findings. Reduced need for phosphate binders and erythropoietin use may lower treatment-related costs. The benefits related to β2-microglobulin and CRP reduction are strongly affected by convective volume. While the findings support the mechanistic rationale for HDF, evidence for the enhanced removal of protein-bound toxins remains limited. Further well-designed studies are needed to determine whether improved solute clearance, especially protein-bound uremic toxins, translates into meaningful long-term clinical outcomes.

## 5. Materials and Methods

### 5.1. Protocol and Registration

We conducted a systematic review and pairwise meta-analysis of randomized controlled trials (RCTs) to evaluate the potential benefits of HDF compared with conventional HD on uremic toxins, inflammatory markers, anemia, and nutritional parameters. The study was performed in accordance with the Preferred Reporting Items for Systematic Reviews and Meta-Analyses (PRISMA) guidelines (Table S8) [61], and the protocol was registered in the PROSPERO database (registration number: CRD420251242804).

### 5.2. Data Sources and Strategy

We searched electronic medical and scientific databases, including PubMed, Scopus, and Cochrane Central Register of Controlled Trials (CENTRAL), to identify relevant literature from their inception dates to 30 November 2025. We used keywords to determine the appropriate controlled vocabulary terms (e.g., MeSH headings). The full search strategy is available in the Supplementary Materials (Table S9). Only studies written in English were included. Two independent authors (W.W., S.J.) screened reference lists of relevant studies and reviews to identify additional eligible articles. Duplicate records were removed using a citation manager and manual review.

### 5.3. Eligibility Criteria

We included RCTs that met the following criteria: (1) enrolled patients aged ≥18 years with ESKD undergoing maintenance HD; (2) compared HDF with conventional HD using low- or high-flux dialyzers; and (3) reported at least one biochemical outcome of interest, including uremic toxins (small, middle, large, or protein-bound), inflammatory biomarkers, anemia-related parameters, or nutritional indicators. Uremic toxins of interest included urea, phosphate, parathyroid hormone, β2-microglobulin, p-cresyl sulfate, and indoxyl sulfate. Inflammatory biomarkers included C-reactive protein (CRP), high-sensitivity CRP (hs-CRP), and interleukin-6 (IL-6). Nutritional status was assessed using serum albumin. Studies were excluded if they were reviews, editorials, conference abstracts, letters or responses, or involved non-human subjects.

### 5.4. Data Extraction and Synthesis

Two authors (W.W. and S.J.) independently screened titles and abstracts, followed by a full-text review of eligible studies. Discrepancies were resolved by consultation with a third reviewer (T.F.). In cases of duplicate reports involving the same patient population, the study with the larger sample size was selected. When studies reported outcomes at multiple time points, data from the longest follow-up period were selected for analysis. Data extraction included the first author, publication year, study country, sample size, patient characteristics, details of HDF and HD prescriptions, dialyzer membrane type, dialysis frequency, outcomes of interest, follow-up duration, and risk-of-bias assessment. For continuous outcomes, mean change values and their standard deviations (SDs) were extracted directly when available. Pre-dialysis plasma levels were selected as they reflect steady-state toxin exposure, in contrast to intradialytic pre–post changes, which are affected by rebound phenomena, solute redistribution, and sampling variability. Baseline (pre-intervention) and endpoint (post-intervention) mean pre-dialysis values with SDs were collected, and pooled baseline means and SDs were further analyzed. When mean changes and SDs were not reported, baseline and end-of-study mean values with corresponding SDs were collected, and mean changes and SDs were calculated in accordance with the Cochrane Handbook for Systematic Reviews of Interventions [62]. If end-of-study values were not explicitly provided, data were derived from graphical presentations of biochemical or other continuous outcomes using the Digitizelt program (http://www.digitizeit.de/).(accessed on 15 December 2025). For studies reporting outcomes as medians with interquartile ranges (IQRs), mean values and SDs were estimated using the methods described by Luo et al. [63] and Wan et al. [64]. For crossover trials, data from the first study period were used when available. When only combined data from both periods were reported, the study was analyzed as a parallel-group trial, with acknowledgment of the potential bias this approach may introduce and cautious interpretation of the results [65].

### 5.5. Risk-of-Bias Assessment

Two authors (W.W. and S.J.) independently evaluated the risk of bias for each study included. Disagreements were resolved through discussion with a third reviewer (T.F.). The methodological quality of the randomized controlled trials was assessed using the Cochrane Risk of Bias 2 (RoB 2) tool [66]. This instrument evaluates five domains: bias arising from the randomization process, deviations from intended interventions, missing outcome data, measurement of outcomes, and selection of the reported results. Each study was categorized as having a low risk of bias, some concerns, or a high risk of bias.

### 5.6. Statistical Analysis

All statistical analyses were performed using Stata version 18 (StataCorp, College Station, TX, USA). A two-tailed *p*-value < 0.05 was considered statistically significant. Meta-analyses were conducted using a random-effects model to generate pooled effect estimates with 95% confidence intervals (CIs). Continuous outcomes were analyzed using weighted mean differences (WMDs) with 95% CIs [67] and were presented alongside mean baseline pre-dialysis plasma levels. Statistical heterogeneity was evaluated using the I^2^ statistic and Cochran’s Q test. The I^2^ statistic ranges from 0% to 100%, with higher values reflecting greater heterogeneity among study results. I^2^ values of 25%, 50%, and 75% or higher represent a low, moderate, and high degree of heterogeneity, respectively. To explore potential sources of heterogeneity, prespecified subgroup analyses were performed based on study design (parallel vs. crossover), geographic region (Asian vs. non-Asian), substitution mode (pre-dilution, post-dilution, or mixed-dilution), intervention duration (≤6 vs. >6 months), and type of dialyzer used in the comparator group (low-flux vs. high-flux). Meta-regression analyses were conducted to assess whether study characteristics, patient factors, or HDF-related variables contributed to between-study heterogeneity. Forest plots were generated to visually assess heterogeneity across studies, and publication bias was evaluated using funnel plots and Egger’s test. Egger’s test was used to complement visual inspection of funnel plots by statistically assessing asymmetry, with a *p*-value < 0.05 indicating potential publication bias.

## Data Availability

No new data were created or analyzed in this study.

## Supplementary Materials

The following supporting information can be downloaded at: https://www.mdpi.com/article/10.3390/toxins18020086/s1; Table S1. Subgroup analyses examining the effect of HDF versus HD on serum phosphorous levels; Table S2. Subgroup analyses examining the effect of HDF versus HD on serum parathyroid hormone levels; Table S3. Subgroup analyses examining the effect of HDF versus HD on serum β2-microglobulin levels; Table S4. Subgroup analyses examining the effect of HDF versus HD on serum CRP levels; Table S5. Subgroup analyses examining the effect of HDF versus HD on serum hemoglobin levels; Table S6. Subgroup analyses examining the effect of HDF versus HD on serum albumin levels; Table S7. Results of meta-regression between variables and biochemical parameters from hemodiafiltration versus hemodialysis; Table S8. PRISMA 2020 checklist Table S9. Search terms; Figure S1. Funnel plot of individual studies illustrating the relationship between standard error and mean difference for the effect of HDF versus HD on serum albumin (*p* = 0.692); Figure S2. Funnel plot of individual studies illustrating the relationship between standard error and mean difference for the effect of HDF versus HD on serum ferritin (*p* = 0.54); Figure S3. Funnel plot of individual studies illustrating the relationship between standard error and mean difference for the effect of HDF versus HD on serum hemoglobin (*p* = 0.72); Figure S4. Funnel plot of individual studies illustrating the relationship between standard error and mean difference for the effect of HDF versus HD on serum interleukin-6 (*p* = 0.08); Figure S5. Funnel plot of individual studies illustrating the relationship between standard error and mean difference for the effect of HDF versus HD on serum β2-microglobulin (*p* = 0.001); Figure S6. Funnel plot of individual studies illustrating the relationship between standard error and mean difference for the effect of HDF versus HD on serum C-reactive protein (*p* = 0.009); Figure S7. Funnel plot of individual studies illustrating the relationship between standard error and mean difference for the effect of HDF versus HD on serum phosphorous (*p* = 0.036).
