# Supplementary material for: Effects of Hemodiafiltration Versus Hemodialysis on Uremic Toxins, Inflammatory Markers, Anemia, and Nutritional Parameters: A Systematic Review and Meta-Analysis
Source: Toxins (Basel). 2026 Feb 6;18(2):86. doi: 10.3390/toxins18020086 (PMC12944842; doi:10.3390/toxins18020086)

Table S1. Sub-group analyses examining the effect of hemodiafiltration versus hemodialysis on serum phosphorous levels.

**Sub-group analyses**	No. of studies	Weighted mean difference (95% CI)	P-values	Assessment of heterogeneity		P for interaction
				I^2 index	P-value	
**Design**						**0.01**
- Crossover	5	-0.57 (-0.76 to -0.38)	<0.01	17.76%	0.30	
- Parallel	13	-0.20 (-0.38 to -0.03)	0.03	92.99%	<0.01	
**Duration**						0.29
- ≤ 6 months	8	-0.41 (-0.71 to -0.12)	0.01	35.90%	0.14	
- > 6 months	10	-0.22 (-0.41 to -0.04)	0.02	95.49%	<0.01	
**Dialyzer**						0.80
- Low flux	4	-0.29 (-0.46 to -0.12)	<0.01	0.00%	0.55	
- High flux	14	-0.26 (-0.44 to -0.07)	0.01	93.78%	<0.01	
**Substitution mode**						0.06
- Post-dilution	14	-0.23 (-0.40 to -0.05)	0.01	87.84%	<0.01	
- Mixed	3	-0.40 (-0.45 to -0.35)	<0.01	0.00%	0.75	
**Race**						0.39
- Asian	2	-0.83 (-2.12 to 0.47)	0.21	37.62%	0.21	
- Non-Asian	16	-0.25 (-0.42 to -0.09)	<0.01	92.70%	<0.01	

Table S2. Sub-group analyses examining the effect of hemodiafiltration versus hemodialysis on serum parathyroid hormone levels.

**Sub-group analyses**	No. of studies	Weighted mean difference (95% CI)	P-values	Assessment of heterogeneity		P for interaction
				I^2 index	P-value	
**Design**						0.36
- Crossover	3	-20.90 (-70.87 to 29.08)	0.41	0.00%	0.92	
- Parallel	9	3.94 (-13.68 to 21.55)	0.66	25.75%	0.21	
**Duration**						0.51
- ≤ 6 months	7	-0.29 (-4.88 to 4.29)	0.90	0.00%	0.51	
- > 6 months	5	9.92 (-20.00 to 39.83)	0.52	25.00%	0.25	
**Dialyzer**						0.67
- Low flux	3	-0.26 (-4.87 to 4.36)	0.91	0.00%	0.71	
- High flux	9	5.40 (-20.42 to 31.21)	0.68	22.43%	0.24	
**Substitution mode**						0.31
- Post-dilution	8	1.90 (-6.30 to 10.11)	0.65	2.76%	0.41	
- Mixed	3	-18.14 (-56.36 to 20.07)	0.35	0.00%	0.93	
**Race**						0.54
- Asian	2	-44.34 (-188.39 to 99.72)	0.55	70.33%	0.07	
- Non-Asian	10	0.21 (-4.31 to 4.73)	0.93	0.00%	0.53	

Table S3. Sub-group analyses examining the effect of hemodiafiltration versus hemodialysis on serum β2-microglobulin levels.

**Sub-group analyses**	No. of studies	Weighted mean difference (95% CI)	P-values	Assessment of heterogeneity		P for interaction
				I^2 index	P-value	
**Design**						**0.01**
- Crossover	5	-9.70 (-14.47 to -4.93)	<0.01	85.91%	<0.01	
- Parallel	11	-3.18 (-4.30 to -2.06)	<0.01	93.45%	<0.01	
**Duration**						**0.04**
- ≤ 6 months	6	-8.74 (-13.58 to -3.89)	<0.01	83.90%	<0.01	
- > 6 months	10	-3.53 (-4.84 to -2.21)	<0.01	96.26%	<0.01	
**Dialyzer**						**<0.01**
- Low flux	5	-11.84 (-15.65 to -8.03)	<0.01	79.48%	<0.01	
- High flux	11	-2.65 (-3.80 to -1.49)	<0.01	94.23%	<0.01	
**Substitution mode**						0.29
- Post-dilution	11	-3.55 (-5.42 to -1.68)	<0.01	94.32%	<0.01	
- Mixed	3	-9.04 (-19.07 to 0.98)	0.08	97.61%	<0.01	

Table S4. Sub-group analyses examining the effect of hemodiafiltration versus hemodialysis on serum CRP levels.

**Sub-group analyses**	No. of studies	Weighted mean difference (95% CI)	P-values	Assessment of heterogeneity		P for interaction
				I^2 index	P-value	
**Design**						0.50
- Crossover	6	-1.47 (-3.36 to 0.43)	0.13	61.38%	0.02	
- Parallel	6	-0.79 (-1.43 to -0.15)	0.02	87.23%	<0.01	
**Duration**						**0.03**
- ≤ 6 months	6	-2.54 (-4.39 to -0.70)	0.01	43.64%	0.11	
- > 6 months	6	-0.45 (-0.99 to 0.09)	0.10	83.94%	<0.01	
**Dialyzer**						0.73
- Low flux	5	-1.32 (-3.09 to 0.45)	0.14	72.61%	0.01	
- High flux	7	-1.78 (-3.66 to 0.10)	0.06	84.94%	<0.01	
**Substitution mode**						0.84
- Post-dilution	9	-0.75 (-1.35 to -0.15)	0.01	82.43%	<0.01	
- Mixed	2	-0.94 (-2.72 to 0.84)	0.30	0.00%	0.66	

Table S5. Sub-group analyses examining the effect of hemodiafiltration versus hemodialysis on serum hemoglobin levels.

**Sub-group analyses**	No. of studies	Weighted mean difference (95% CI)	P-values	Assessment of heterogeneity		P for interaction
				I^2 index	P-value	
**Design**						0.90
- Crossover	4	0.08 (-0.02 to 0.18)	0.11	0.00%	0.72	
- Parallel	10	0.07 (-0.09 to 0.23)	0.40	87.60%	<0.01	
**Duration**						0.12
- ≤ 6 months	5	-0.22 (-0.64 to 0.19)	0.30	68.57%	0.01	
- > 6 months	9	0.12 (-0.01 to 0.25)	0.06	86.32%	<0.01	
**Dialyzer**						0.76
- Low flux	3	0.09 (-0.05 to 0.23)	0.22	0.00%	0.94	
- High flux	11	0.06 (-0.09 to 0.20)	0.46	86.57%	<0.01	
**Substitution mode**						0.26
- Post-dilution	12	0.08 (-0.06 to 0.22)	0.25	84.95%	<0.01	
- Mixed	2	-0.08 (-0.31 to 0.16)	0.53	26.02%	0.24	

Table S6. Sub-group analyses examining the effect of hemodiafiltration versus hemodialysis on serum albumin levels.

**Sub-group analyses**	No. of studies	Weighted mean difference (95% CI)	P-values	Assessment of heterogeneity		P for interaction
				I^2 index	P-value	
**Design**						0.09
- Crossover	6	-0.10 (-0.13 to -0.06)	<0.01	0.00%	0.67	
- Parallel	9	-0.03 (-0.10 to 0.04)	0.45	79.46%	<0.01	
**Duration**						0.08
- ≤ 6 months	8	-0.10 (-0.13 to -0.06)	<0.01	0.00%	0.77	
- > 6 months	7	-0.02 (-0.10 to 0.06)	0.63	83.67%	<0.01	
**Dialyzer**						0.39
- Low flux	7	-0.08 (-0.14 to -0.01)	0.02	70.31%	<0.01	
- High flux	8	-0.03 (-0.12 to 0.05)	0.46	79.60%	<0.01	
**Substitution mode**						0.91
- Post-dilution	12	-0.05 (-0.10 to 0.01)	0.08	77.33%	<0.01	
- Mixed	2	-0.05 (-0.17 to 0.06)	0.36	38.54%	0.20	

Table S7. Results of meta-regression between variables and biochemical parameters from hemodiafiltration versus hemodialysis.

Variable	Beta	SE	95% CI	z	P-value	I^2 (%)	R^2 (%)
**Serum β2-microglobulin**							
Convection volume	-0.165	0.078	-0.318 to -0.013	-2.13	0.033	95.70	0.00
Dialysis vintage	-0.015	0.040	-0.092 to 0.063	-0.37	0.714	95.12	0.00
Age	0.306	0.143	0.259 to 0.587	2.14	0.032	94.46	0.00
Publication year	0.249	0.121	0.118 to 0.487	2.06	0.040	94.71	0.00
Sample size	0.006	0.002	0.002 to 0.011	2.65	0.008	95.21	0.00
**Serum phosphorous**							
Convection volume	0.029	0.024	-0.018 to 0.077	0.01	0.990	90.36	24.52
Dialysis vintage	0.005	0.005	-0.005 to 0.014	0.97	0.333	89.31	0.00
Age	0.009	0.013	-0.017 to 0.034	0.69	0.492	90.14	0.00
Publication year	0.014	0.013	-0.012 to 0.040	1.04	0.300	90.91	5.42
Sample size	<0.001	<0.001	<-0.001 to 0.001	1.32	0.186	92.38	0.00
**Serum parathyroid hormone**							
Convection volume	0.029	2.434	-4.742 to 4.800	-0.07	0.941	2.15	0.00
Dialysis vintage	-0.157	0.440	-1.019 to 0.705	-0.36	0.721	13.80	0.00
Age	-0.565	1.451	-3.408 to 2.278	-0.39	0.697	12.37	0.00
Publication year	0.244	2.870	-5.380 to 5.869	0.09	0.932	13.30	0.00
Sample size	0.049	0.027	-0.004 to 0.102	1.80	0.072	0.00	100.00
**Serum C-reactive protein**							
Convection volume	-0.301	0.126	-0.547 to -0.055	-2.40	0.017	82.47	0.00
Dialysis vintage	-0.045	0.021	-0.087 to -0.004	-2.14	0.033	79.67	0.00
Age	0.284	0.186	-0.081 to 0.648	1.53	0.127	81.11	0.00
Publication year	0.026	0.102	-0.174 to 0.225	0.25	0.801	81.30	0.00
Sample size	0.002	0.001	<-0.001 to 0.005	1.83	0.067	80.78	0.00
**Serum albumin**							
Convection volume	-0.011	0.010	-0.032 to 0.009	-1.10	0.272	72.74	0.25
Dialysis vintage	0.001	0.002	-0.003 to 0.005	0.50	0.614	78.69	0.00
Age	-0.001	0.005	-0.010 to 0.008	-0.18	0.854	75.79	0.00
Publication year	0.008	0.005	-0.002 to 0.018	1.64	0.101	70.30	2.18
Sample size	<0.001	<0.001	<-0.001 to <0.001	0.19	0.848	75.25	0.00

Table S8. PRISMA 2020 Checklist

Section and Topic	Item #	Checklist item	Location where item is reported
**TITLE**			
Title	1	Identify the report as a systematic review.	1
**ABSTRACT**			
Abstract	2	See the PRISMA 2020 for Abstracts checklist.	1
**INTRODUCTION**			
Rationale	3	Describe the rationale for the review in the context of existing knowledge.	2
Objectives	4	Provide an explicit statement of the objective(s) or question(s) the review addresses.	2
**METHODS**			
Eligibility criteria	5	Specify the inclusion and exclusion criteria for the review and how studies were grouped for the syntheses.	17
Information sources	6	Specify all databases, registers, websites, organizations, reference lists and other sources searched or consulted to identify studies. Specify the date when each source was last searched or consulted.	17 and references
Search strategy	7	Present the full search strategies for all databases, registers and websites, including any filters and limits used.	17, Table S8
Selection process	8	Specify the methods used to decide whether a study met the inclusion criteria of the review, including how many reviewers screened each record and each report retrieved, whether they worked independently, and if applicable, details of automation tools used in the process.	17-18
Data collection process	9	Specify the methods used to collect data from reports, including how many reviewers collected data from each report, whether they worked independently, any processes for obtaining or confirming data from study investigators, and if applicable, details of automation tools used in the process.	17
Data items	10a	List and define all outcomes for which data were sought. Specify whether all results that were compatible with each outcome domain in each study were sought (e.g., for all measures, time points, analyses), and if not, the methods used to decide which results to collect.	17-18
	10b	List and define all other variables for which data were sought (e.g., participant and intervention characteristics, funding sources). Describe any assumptions made about any missing or unclear information.	17-18
Study risk of bias assessment	11	Specify the methods used to assess risk of bias in the included studies, including details of the tool(s) used, how many reviewers assessed each study and whether they worked independently, and if applicable, details of automation tools used in the process.	18
Effect measures	12	Specify for each outcome the effect measure(s) (e.g., risk ratio, mean difference) used in the synthesis or presentation of results.	18
Synthesis methods	13a	Describe the processes used to decide which studies were eligible for each synthesis (e.g., tabulating the study intervention characteristics and comparing against the planned groups for each synthesis (item #5)).	17-18
	13b	Describe any methods required to prepare the data for presentation or synthesis, such as handling of missing summary statistics, or data conversions.	17-18
	13c	Describe any methods used to tabulate or visually display results of individual studies and syntheses.	18
	13d	Describe any methods used to synthesize results and provide a rationale for the choice(s). If meta-analysis was performed, describe the model(s), method(s) to identify the presence and extent of statistical heterogeneity, and software package(s) used.	18
	13e	Describe any methods used to explore possible causes of heterogeneity among study results (e.g., sub-group analysis, meta-regression).	18

Section and Topic	Item #	Checklist item	Location where item is reported
	13f	Describe any sensitivity analyses conducted to assess robustness of the synthesized results.	18
Reporting bias assessment	14	Describe any methods used to assess the risk of bias due to missing results in a synthesis (arising from reporting biases).	18
Certainty assessment	15	Describe any methods used to assess certainty (or confidence) in the body of evidence for an outcome.	NA
**RESULTS**			
Study selection	16a	Describe the results of the search and selection process, from the number of records identified in the search to the number of studies included in the review, ideally using a flow diagram.	2-3, Figure 1
	16b	Cite studies that might appear to meet the inclusion criteria, but which were excluded, and explain why they were excluded.	2-3, Figure 1
Study characteristics	17	Cite each included study and present its characteristics.	4-7, Table 1, 2
Risk of bias in studies	18	Present assessments of risk of bias for each included study.	8, Figure 2A, 2B
Results of individual studies	19	For all outcomes, present, for each study: (a) summary statistics for each group (where appropriate) and (b) an effect estimate and its precision (e.g., confidence/credible interval), ideally using structured tables or plots.	9-13 Figure 3-6 Table 3
Results of syntheses	20a	For each synthesis, briefly summarize the characteristics and risk of bias among contributing studies.	2-9, Table 1
	20b	Present results of all statistical syntheses conducted. If meta-analysis was done, present for each the summary estimate and its precision (e.g. confidence/credible interval) and measures of statistical heterogeneity. If comparing groups, describe the direction of the effect.	9-13 Figure 3-6 Table 3
	20c	Present results of all investigations of possible causes of heterogeneity among study results.	9-14 Figure 7 Table S1-S7
	20d	Present results of all sensitivity analyses conducted to assess the robustness of the synthesized results.	Table S1-S7
Reporting biases	21	Present assessments of risk of bias due to missing results (arising from reporting biases) for each synthesis assessed.	12, Figure 2
Certainty of evidence	22	Present assessments of certainty (or confidence) in the body of evidence for each outcome assessed.	NA
**DISCUSSION**			
Discussion	23a	Provide a general interpretation of the results in the context of other evidence.	11
	23b	Discuss any limitations of the evidence included in the review.	11-17
	23c	Discuss any limitations of the review processes used.	11-17

Section and Topic	Item #	Checklist item	Location where item is reported
	23d	Discuss implications of the results for practice, policy, and future research.	16-17
**OTHER INFORMATION**			
Registration and protocol	24a	Provide registration information for the review, including the register name and registration number, or state that the review was not registered.	17
	24b	Indicate where the review protocol can be accessed, or state that a protocol was not prepared.	17
	24c	Describe and explain any amendments to information provided at registration or in the protocol.	N/A
Support	25	Describe sources of financial or non-financial support for the review, and the role of the funders or sponsors in the review.	19
Competing interests	26	Declare any competing interests of review authors.	19
Availability of data, code and other materials	27	Report which of the following are publicly available and where they can be found: template data collection forms; data extracted from included studies; data used for all analyses; analytic code; any other materials used in the review.	19

From: Page MJ, McKenzie JE, Bossuyt PM, Boutron I, Hoffmann TC, Mulrow CD, et al. The PRISMA 2020 statement: an updated guideline for reporting systematic reviews. BMJ 2021;372:n71. doi: 10.1136/bmj.n71

Table S9. Search terms

Keywords	No. of articles
Pubmed	**395**
Population of interest<br>("Kidney Failure, Chronic"[Mesh] OR end stage kidney disease[tiab] OR end stage renal disease[tiab] OR ESKD[tiab] OR ESRD[tiab])<br>**AND**<br>*Intervention of interest*<br>("Hemodiafiltration"[Mesh] OR hemodiafiltration [tiab] OR haemodiafiltration [tiab] OR HDF[tiab]) AND ("Renal Dialysis"[Mesh] OR hemodialysis[tiab] OR haemodialysis[tiab] OR HD[tiab]<br>OR dialysis[tiab])<br>**AND**<br>*Study design of interest*<br>((“randomized controlled trial”[Publication Type] OR “controlled clinical trial” [Publication Type] OR “randomized”[All Fields] OR “placebo”[Title/Abstract] OR “drug therapy”[MeSH Subheading] OR “randomly”[Title/Abstract] OR “trial”[Title/Abstract] OR “groups”[Title/Abstract] NOT (“animals”[MeSH Terms] NOT “humans”[MeSH Terms]))	
Scopus	**367**
Population of interest<br>TITLE-ABS-KEY<br>(end stage kidney disease OR end stage renal disease OR ESKD OR ESRD)<br>**AND**<br>*Intervention of interest*<br>(TITLE-ABS-KEY (hemodiafiltration OR haemodiafiltration OR HDF)<br>AND<br>TITLE-ABS-KEY (hemodialysis OR haemodialysis OR HD OR dialysis)<br>**AND**<br>*Study design of interest*<br>TITLE-ABS-KEY ("randomized controlled trial" OR "controlled clinical trial" OR "randomized" OR "placebo" OR "drug<br>therapy" OR "randomly" OR "trial" OR "groups"))	
CENTRAL	**192**
Population of interest<br>“end stage kidney disease” OR “end stage renal disease” OR “ESKD” OR “ESRD”<br>**AND**<br>*Intervention of interest*<br>hemodiafiltration OR haemodiafiltration OR HDF AND hemodialysis OR haemodialysis OR HD OR dialysis<br>**AND**	

Study design of interest<br>"randomized controlled trial" OR "controlled clinical trial" OR "randomized" OR "placebo" OR "drug therapy" OR "randomly" OR "trial" OR "groups"	

Figure S1. Funnel plot of individual studies illustrating the relationship between standard error and mean difference for the effect of HDF versus HD on serum albumin (P = 0.692).

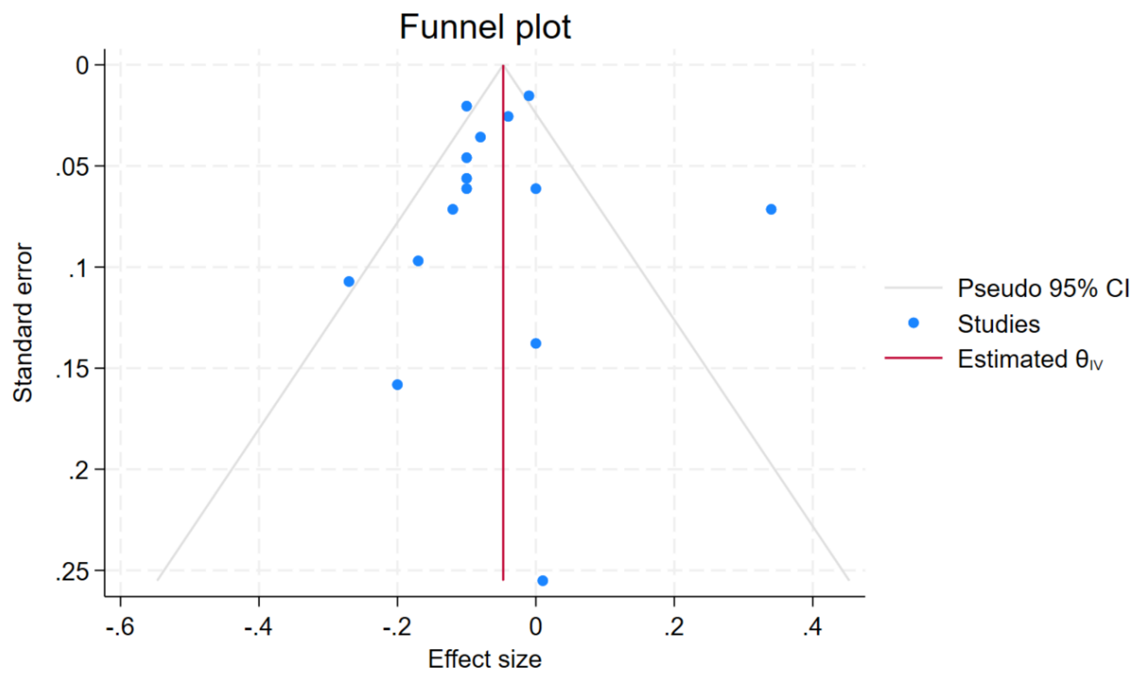

Figure S2. Funnel plot of individual studies illustrating the relationship between standard error and mean difference for the effect of HDF versus HD on serum ferritin (P = 0.54).

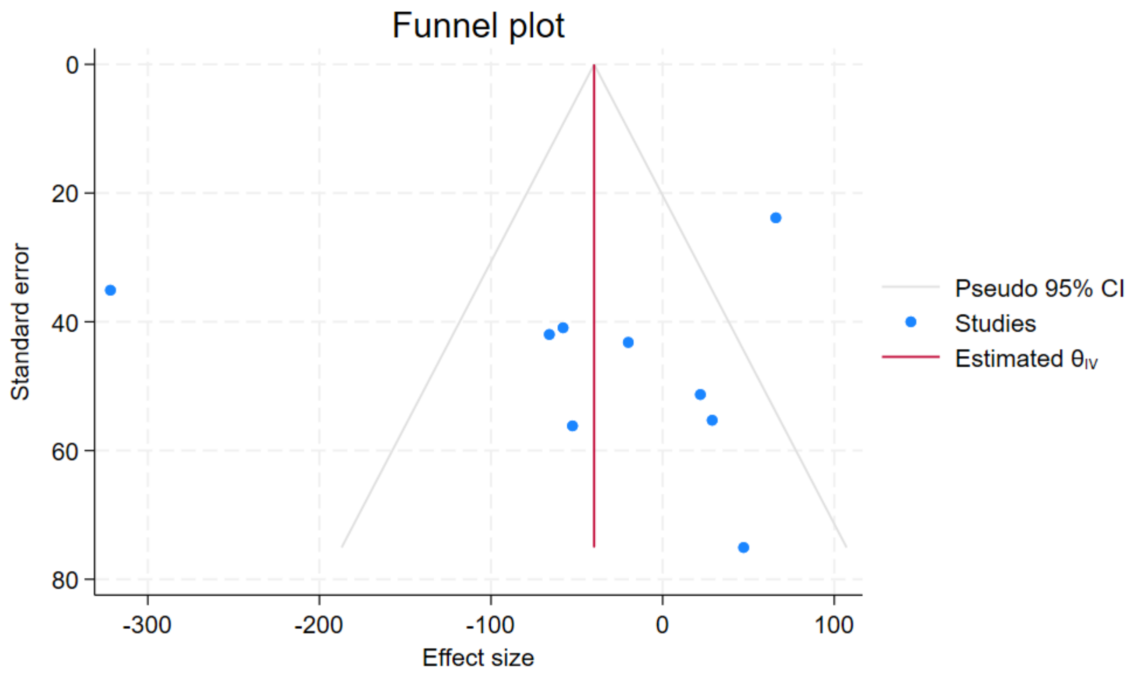

Figure S3. Funnel plot of individual studies illustrating the relationship between standard error and mean difference for the effect of HDF versus HD on serum hemoglobin (P = 0.72).

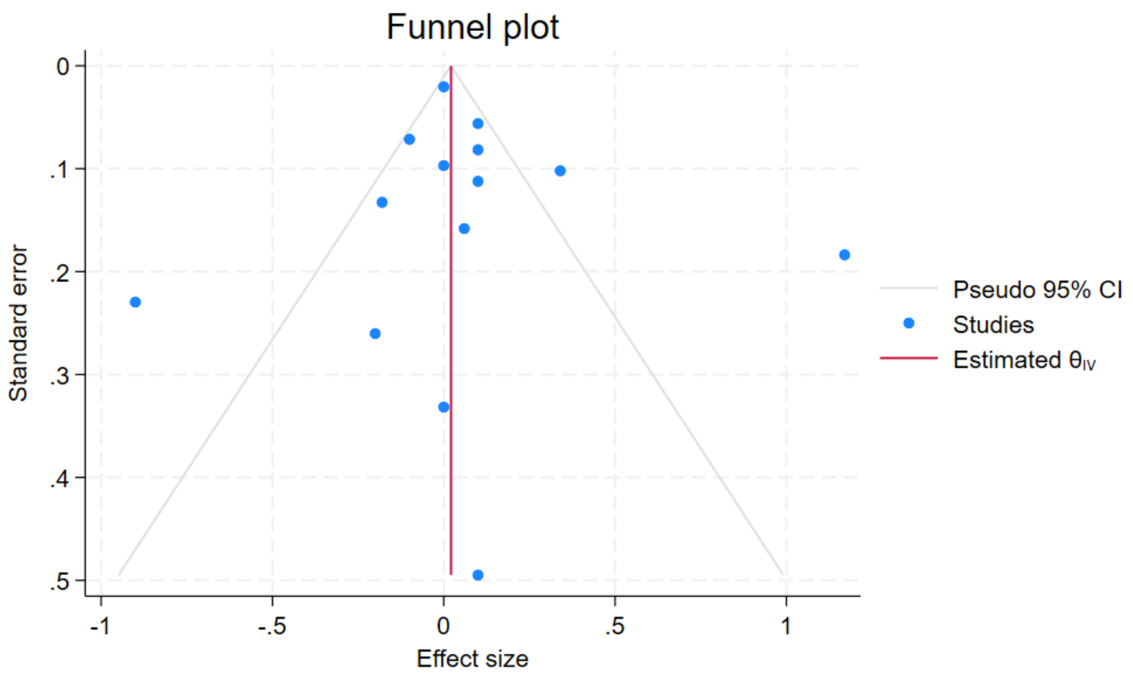

Figure S4. Funnel plot of individual studies illustrating the relationship between standard error and mean difference for the effect of HDF versus HD on serum interleukin-6 (P = 0.08).

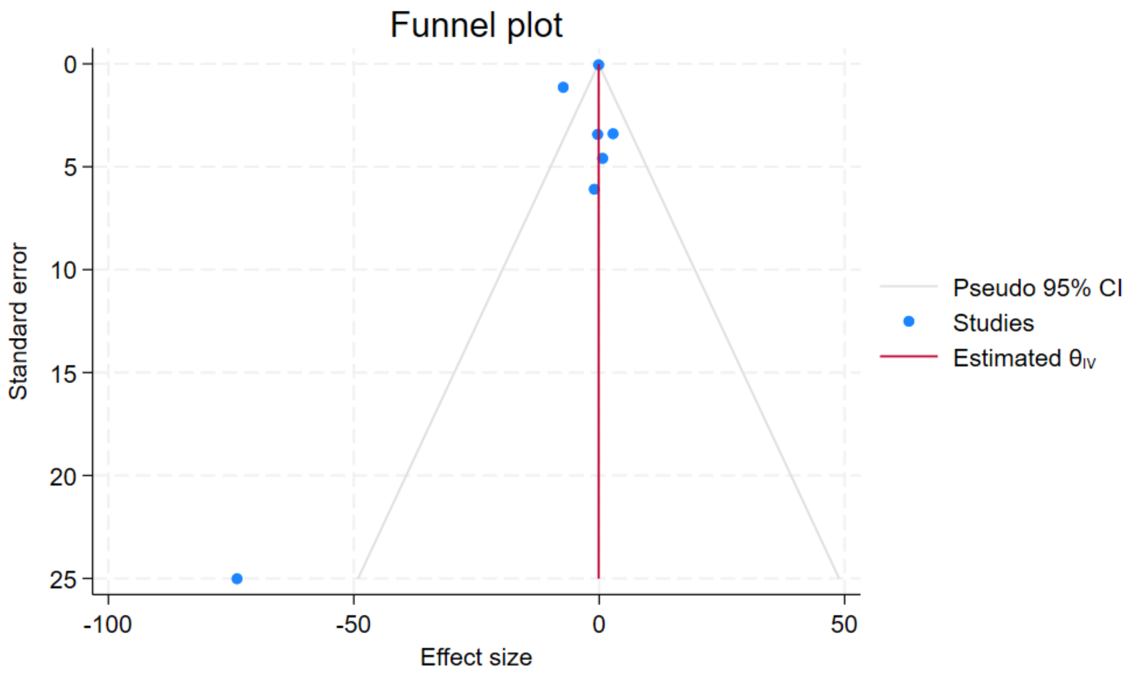

Figure S5. Funnel plot of individual studies illustrating the relationship between standard error and mean difference for the effect of HDF versus HD on serum β2-microglobulin (P = 0.001).

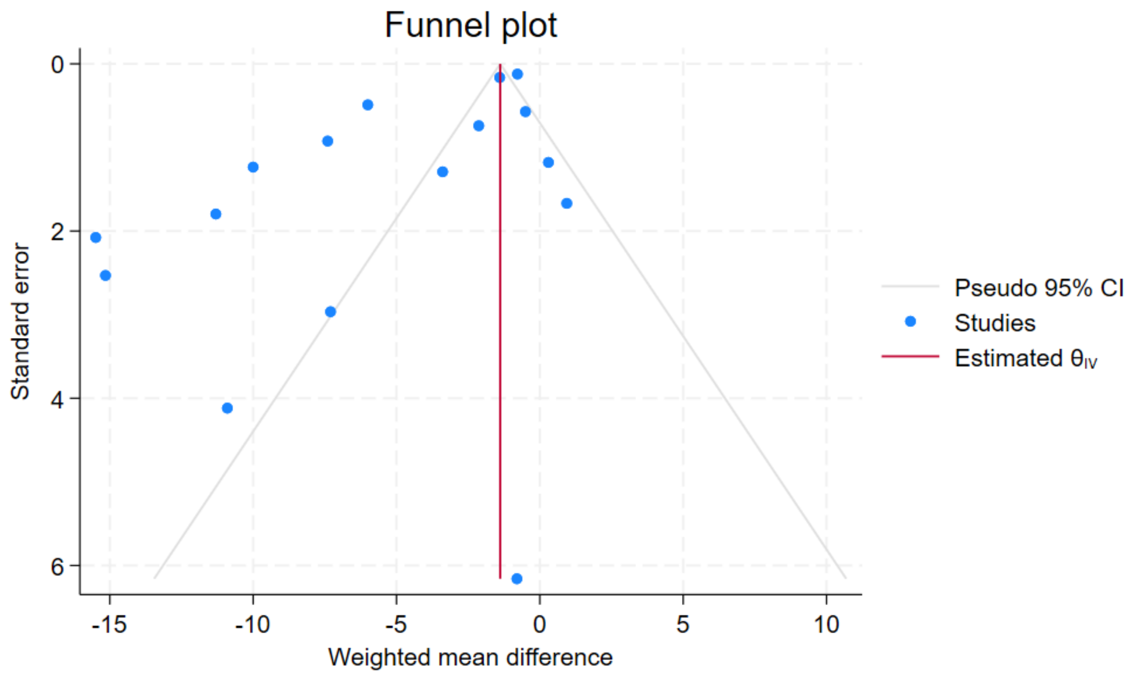

Figure S6. Funnel plot of individual studies illustrating the relationship between standard error and mean difference for the effect of HDF versus HD on serum C-reactive protein (P = 0.009).

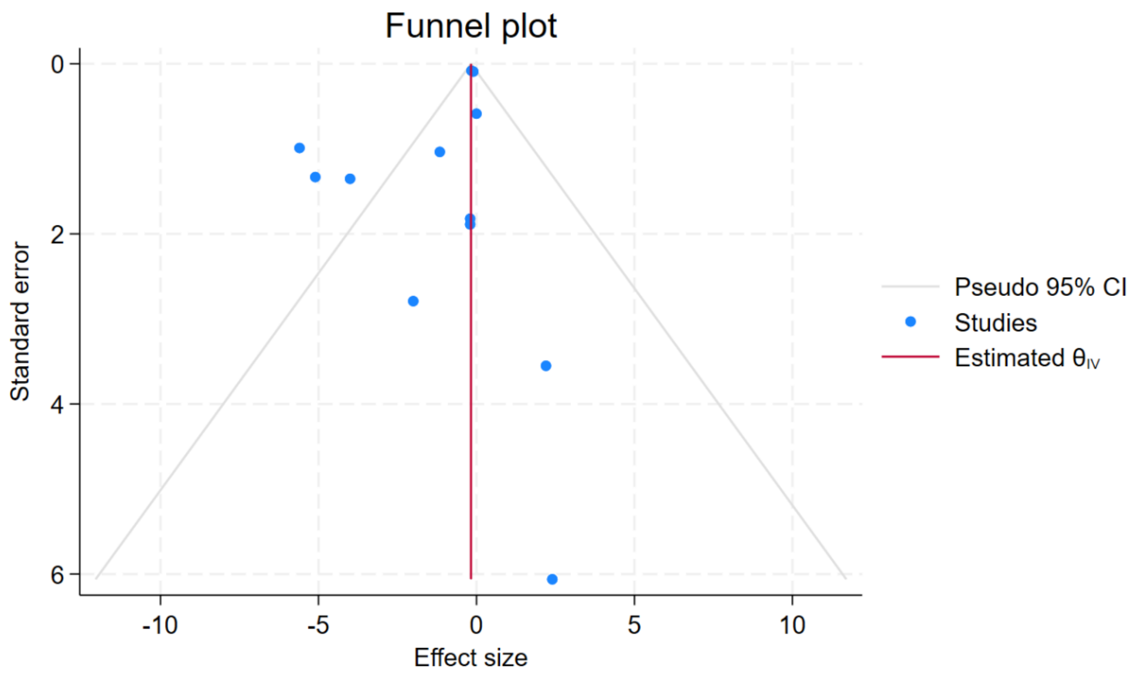

Figure S7. Funnel plot of individual studies illustrating the relationship between standard error and mean difference for the effect of HDF versus HD on serum phosphorous (P = 0.036).

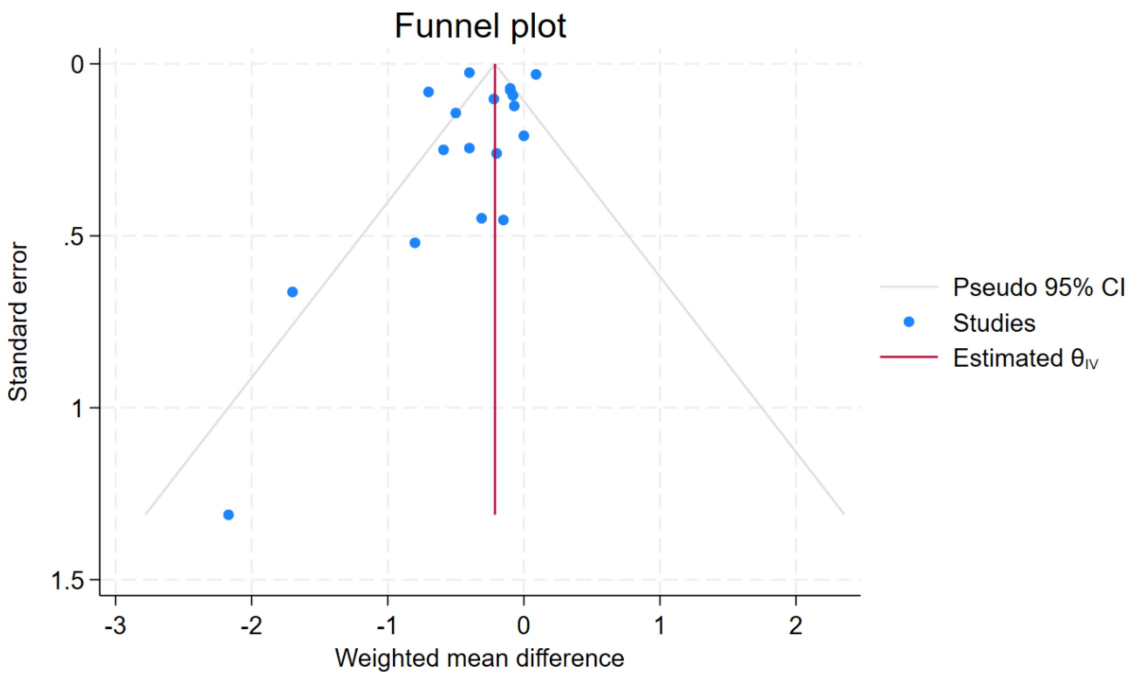

Supplement: Supplementary file 1 [file toxins-18-00086-s001.zip › toxins-4102883-supplementary.pdf]
